# Supplementary material for: Dynamics of temporal influence in polarised networks
Source: PLoS One. 2025 Dec 9;20(12):e0337753. doi: 10.1371/journal.pone.0337753 (PMC12688149; doi:10.1371/journal.pone.0337753)
Supplement: S1 Appendix — (PDF) [file pone.0337753.s001.pdf]

S1 Appendix. Randomisation of networks with communities

Newman et al. [1] investigated network properties, such as clustering coefficient, average degree and shortest paths of random networks with arbitrary degree distributions. They showed that the random graph models constructed from real networks perform well in estimating quantities investigated, and in some cases give results of high accuracy. Creating a random network that maintains the degree distribution of the original network is a common practice among network researchers [2, 3] as it simplifies the network structure while still giving good estimates on the network properties.

The configuration model [4] is a flexible and powerful type of random network that may take any degree sequence as we please [5], where the exact degree of each node is specified through in-stubs — the number of edges ending on the node — and out-stubs — the number of edges starting on the node.

When it comes to networks with communities, such as the Twitter Irish Abortion Referendum network here studied, various types of stubs must be considered. We need to account for not only in-stubs and out-stubs coming from (going to) the same community — the in-community stubs — but also for in-stubs and out-stubs between communities — the inter-communities stubs. Our Twitter network, which we will be referring to as RT8 network [6], has two communities, therefore four types of stubs must be considered for each node: the in-community in-stubs and out-stubs, and the inter-communities in-stubs and out-stubs. To create the random network, we first create each community separately, each one containing nodes connected through in-community stubs. We then connect the communities by using the inter-community stubs, where a inter-community out-stub of a node in community 1 is connected to the inter-community in-stub of a node in community 2, and vice-versa.

References

1. Newman ME, Strogatz SH, Watts DJ. Random graphs with arbitrary degree distributions and their applications. Physical review E. 2001;64(2):026118.

2. Kivelä M, Pan RK, Kaski K, Kertész J, Saramäki J, Karsai M. Multiscale analysis of spreading in a large communication network. Journal of Statistical Mechanics: Theory and Experiment. 2012;2012(03):P03005.

3. Karsai M, Kivelä M, Pan RK, Kaski K, Kertész J, Barabási AL, et al. Small but slow world: How network topology and burstiness slow down spreading. Physical Review E—Statistical, Nonlinear, and Soft Matter Physics. 2011;83(2):025102.

4. Bollobás B. A probabilistic proof of an asymptotic formula for the number of labelled regular graphs. European Journal of Combinatorics. 1980;1(4):311–316.

5. Newman M. Networks. Oxford university press; 2018.

6. Pena CB, MacCarron P, O'Sullivan DJ. Finding polarized communities and tracking information diffusion on Twitter: a network approach on the Irish Abortion Referendum. Royal Society Open Science. 2025;12(1):240454.
